# Recounting a Common Experience: On the Effectiveness of Instructing Eyewitness Pairs

**DOI:** 10.3389/fpsyg.2018.00284

**Published:** 2018-03-09

**Authors:** Annelies Vredeveldt, Peter J. van Koppen

**Affiliations:** Department of Criminal Law and Criminology, Vrije Universiteit Amsterdam, Amsterdam, Netherlands

**Keywords:** eyewitness memory, investigative interviewing, collaborative recall, retrieval strategy, conformity

## Abstract

Pairs of eyewitnesses with a content-focused interaction style remember significantly more about witnessed incidents. We examined whether content-focused retrieval strategies can be taught. Seventy-five pairs of witnesses were interviewed thrice about an event. The first and third interview were conducted individually for all witnesses. The second interview was individual, collaborative without instruction, or collaborative with instruction. Pairs in the latter condition were instructed to actively listen to and elaborate upon each other's contributions. The strategy instruction had no effect on retrieval strategies used, nor on the amount or accuracy of reported information. However, pairs who spontaneously adopted a content-focused interaction style during the collaborative interview remembered significantly more. Thus, our findings show that effective retrieval strategies cannot be taught, at least not with the current instructions. During the second interview, we observed collaborative inhibition and error pruning. When considering the total amount of information reported across the first two interviews, however, collaboration had no inhibitory effect on correct recall, yet the error pruning benefits remained. These findings suggest that investigative interviewers should interview witnesses separately first, and then interview pairs of witnesses collaboratively.

## Introduction

In the past two decades, legal psychologists have recommended that eyewitnesses should be prevented from talking to each other. This recommendation is based on a series of studies showing that witnesses can contaminate each other's memory, a phenomenon known as memory conformity (Wright et al., 2000; Gabbert et al., 2003) or social contagion (Roediger et al., 2001; Meade and Roediger, 2002). For example, Gabbert and colleagues found that after discussion with a co-witness, 71% of witnesses reported items about a witnessed event that they could not possibly have seen, because the event they saw did not contain those items. Although memory conformity studies have highlighted an important danger of discussion between witnesses, they have not examined the possibility that co-witness discussion can also have benefits. In the present study, we explored whether potential benefits of co-witness discussion can be maximized by instructing witnesses to collaborate effectively.

Recent research on collaborative eyewitness interviews shows that discussion between witnesses does have benefits (Vredeveldt et al., 2016a, 2017). In those studies, collaboration between two witnesses resulted in more accurate testimony. The error pruning effect observed in collaborative eyewitness interviews is in line with findings on collaborative recall of simple stimuli, which shows that collaboration typically leads to more accurate reporting (e.g., Ross et al., 2004; Harris et al., 2012; Hyman et al., 2013). Thus, although it is certainly possible that witnesses adopt each other's errors, as the memory conformity literature shows, this is not the whole story. When two individuals who have witnessed the same event participate in a collaborative interview, they may actually be more likely to prune each other's errors than to adopt each other's errors (Vredeveldt et al., 2017).

In addition to the accuracy of reported information, the amount of information in eyewitness memory reports was assessed (Vredeveldt et al., 2016a, 2017). On average, collaboration did not increase or reduce the amount of correct information reported about the witnessed event. This finding is surprising in light of the robust collaborative inhibition effect typically reported in the collaborative recall literature. Collaborative inhibition refers to the finding that a group of individuals recalling together (i.e., a collaborative group) remembers less than the same number of individuals recalling on their own (i.e., a nominal group; Weldon and Bellinger, 1997). This occurs because partners disrupt each other's individual strategies to retrieve information from memory (e.g., the order in which to recall a set of learned words) and because one person's utterances during the discussion can inhibit the partner's retrieval of different items from memory (see Basden et al., 1997; Hyman et al., 2013; Barber et al., 2015). Collaborative inhibition has generally been found for recall of simple stimuli (see Marion and Thorley, 2016, for an overview), but more recently also for written reports about witnessed events (Yaron-Antar and Nachson, 2006; Wessel et al., 2015; Bärthel et al., 2017). The finding that collaborative inhibition is not observed in collaborative eyewitness interviews could potentially be explained by the retrieval cues that the interviewer provides throughout the interview. Thus, the interviewer's follow-up questions about the specifics of the event may reduce the likelihood that witnesses forget elements due to strategy disruption or retrieval inhibition.

So far, we have only discussed *average* differences in the amount of reported information between collaborative and nominal groups. Importantly, however, there is considerable variability in the effect of collaboration on the amount reported. Whereas, collaboration inhibits recall in some groups, it facilitates recall in other groups. For example, some married couples help each other remember more about autobiographical events (Harris et al., 2011, 2014), some groups of air plane pilots facilitate each other's recall of aviation scenarios (Meade et al., 2009), some pairs of police officers help each other write more complete police reports (Vredeveldt et al., 2016b) and some pairs of witnesses facilitate each other's recall of a witnessed incident (Vredeveldt et al., 2016a, 2017). What successful pairs seem to have in common, is that they use particularly effective collaborative retrieval strategies. In all of the above-mentioned studies, analyses of the discussion between partners revealed that those who actively listen to each other's contributions (e.g., by repeating or rephrasing what the partner has said) and build upon each other's contributions (e.g., by elaborating with relevant information) facilitate each other's recall. Principal component analysis conducted by Vredeveldt et al. (2016a) revealed that acknowledgments, repetitions, reformulations and elaborations frequently occur together. They coined the term *content-focused interaction* to refer to this style of acknowledging, repeating, rephrasing and elaborating upon the partner's contributions.

For other types of collaborative retrieval strategies, findings have been more mixed. For example, corrections of the partner's statements have been associated with both increased (Meade et al., 2009) and decreased (Harris et al., 2011) memory output. Attempts at cuing each other's memory increased memory output in one study (Harris et al., 2011) and explanations of one's own statements increased memory output in another study (Meade et al., 2009). Principal component analysis (Vredeveldt et al., 2016a) revealed that corrections, explanations, successful and failed cuing attempts, references to the relationship, and expressions of renewed remembering frequently occur together. This set of strategies is somewhat less coherent than content-focused interaction component, but has been conceptualized as *process-focused interaction* (Vredeveldt et al., 2016a), because most of these strategies seem more concerned with the process of remembering together than with the content of the partner's contributions. This component has not been associated with collaborative benefits or costs in eyewitness interview studies (Vredeveldt et al., 2016a,b, 2017). In sum, whereas content-focused retrieval strategies have consistently been associated with a greater amount of information recalled, process-focused strategies typically do not benefit recall.

Given that content-focused retrieval strategies are so effective, it may be beneficial to encourage eyewitnesses to use them in collaborative interviews. Therefore, we explored the potential value of a strategy instruction at the start of a collaborative eyewitness interview. In the present study, 75 pairs of witnesses assigned to one of three experimental conditions were interviewed three times about a videotaped event. In legal settings, it is crucial to obtain independent witness reports before allowing witnesses to talk to each other; hence all participants were first interviewed individually. During the second interview, participants in the nominal condition were again interviewed individually, whereas participants in two collaborative conditions recalled the event in pairs. At the start of the second interview, pairs in the collaborative-instruction condition were instructed on how to remember together effectively, whereas pairs in the collaborative-none condition received no such instruction. The final interview was again individual in all conditions, to allow for an analysis of post-collaborative individual memory (cf. e.g., Bärthel et al., 2017).

Based on previous findings, we hypothesized that encouraging witnesses to use content-focused retrieval strategies would increase the amount of information they reported. Nonetheless, we also considered the possibility that content-focused interaction strategies are effective only when partners adopt them naturally, not when they are forced to do so. Moreover, it may not be possible at all to influence partners' retrieval strategies with a simple instruction.

## Methods

This research was preregistered via the Open Science Framework, available at https://osf.io/t8hyc/.

### Participants and design

Power calculations were conducted based on previous studies in which pairs of witnesses were interviewed collaboratively about a witnessed event (Vredeveldt et al., 2016a, 2017). Because those studies revealed no significant differences between collaborating pairs and non-collaborating pairs in correct recall, we selected reported effect sizes for errors: *d* = −0.80 and *d* = −0.91, respectively. A sample size of 25 pairs per condition (i.e., 75 pairs in total) would allow us to detect an effect of *d* = 0.80 with power = 0.80 at the standard 0.05 alpha error probability.

We recruited 150 participants (111 female, 39 male), with ages ranging from 18 to 79 (*M* = 27.41; *SD* = 12.98). All participants were fluent in Dutch and 79% were students. Participants gave written informed consent prior to participating. In accordance with the guidelines of the VU Faculty of Law, assessment by the Ethics Committee for Legal and Criminological Research was not required for this study. Participants were randomly coupled with an experimental partner, whom they did not know prior to the study. Pairs were randomly assigned to one of three conditions: nominal, collaborative without strategy instruction (collaborative-none) or collaborative with strategy instruction (collaborative-instruction).

There was no significant difference in the gender composition of pairs in the nominal condition (2 both male, 12 both female, 11 mixed), the collaborative-none condition (1 both male, 16 both female, 8 mixed) and the collaborative-instruction condition (2 both male, 13 both female, 10 mixed), $\chi_{\left(4\right)}^{2}$ = 1.52, *p* = 0.824, Cramer's *V* = 0.10. Because the age distribution was extremely positively skewed (*Z* = 10.98, *p* < 0.001) and leptokurtic (*Z* = 9.81, *p* < 0.001) and could not be transformed into a normal distribution, we conducted non-parametric tests to assess differences between conditions. Kruskall-Wallis tests revealed a significant age difference between conditions, *H*_(2)_ = 11.21, *p* = 0.004. Follow-up Mann-Whitney tests showed that participants in the collaborative-none condition were significantly younger (*Mdn* = 21, *M* = 23.74, *SD* = 9.10) compared to the nominal condition (*Mdn* = 23, *M* = 28.06, *SD* = 13.43), *U* = 826.00, *p* = 0.002, and the collaborative-instruction condition (*Mdn* = 24, *M* = 30.44, *SD* = 15.03), *U* = 840.00, *p* = 0.005, with no significant difference between the latter two conditions, *U* = 1184.50, *p* = 0.651. The age difference will be addressed in the Discussion.

### Materials

Participants watched a 70-s video clip taken from a relatively unknown Dutch TV series. At the start of the clip, a woman walks onto the street and almost gets run over by a car. She is pushed out of the way just in time by a man; they both land on the ground. The car then chases the man and the woman through the streets, until they enter a narrow alleyway. While standing in the alleyway, they see the tinted car window roll down slightly, revealing a threatening hand gesture of a shooting gun.

### Procedure

Participants were recruited through advertisements in local newspapers and flyers at the local university and in the surrounding neighborhood for participation in a study on “criminal behavior.” They were paid €10 for their participation. Two participants per session arrived at the laboratory. None of the pair members knew each other prior to participating. Upon arrival, pair members were seated in the same room, signed an informed consent form and watched the video together, with a researcher present in the room. They were instructed not to speak to each other during or after the video. After watching the video, participants were guided to separate rooms and asked to individually complete a 5-min word-finder distracter task.

All participants were interviewed three times about the video. Two interviewers blind to the study's hypotheses conducted all interviews. In the nominal condition, all interviews were conducted individually. Participants in the nominal condition remained in the same room for all three interviews, while the two interviewers switched rooms: each participant was interviewed by the same interviewer during the first and third interview but by a different interviewer during the second interview. This was done to avoid potential reluctance on the part of the participant to tell the same story thrice to the same interviewer (see also Shaw et al., 2014). In the collaborative conditions, the first and third interviews were conducted individually; the second interview was conducted collaboratively. The two individual interviews were conducted by two different interviewers and the collaborative interview was conducted by one of those two interviewers.

At the start of each interview, participants were instructed to remember as much as possible, but not to guess. They were asked to tell the interviewer if they did not know the answer to a question. Each interview followed a strict interview protocol, modeled after the protocol used by Dutch police interviewers (Van Amelsvoort et al., 2015). It consisted of four phases: free recall, follow-up questions tailored to the participant's testimony, specific questions about the people in the scene and specific questions about the context in which the events took place. In the second and third interview, participants were instructed to tell their full story again and assume that the interviewer did not know what they had said during the previous interview.

At the start of the second interview, participants in the collaborative-none condition did not receive any instruction on how to collaborate. Participants in the collaborative-instruction condition received the following instruction (translated from Dutch):

Please work together to give an account that is as complete as possible and try to help each other remember. Previous research shows that partners who repeat and elaborate upon each other's statements, remember more together. In the interview that follows, please listen carefully to each other's contributions. You can also regularly repeat what the other person says or try to summarize it in your own words. Try to build upon what your partner says by adding new information. We do not want an interview in which one of you tells their story first and only then the other person starts speaking—make sure you leave breaks so that there is room for the other person to add to what you are saying. Is this all clear?

All interviews were audio-recorded. At the end of the session, participants provided demographic information. Finally, participants were debriefed and thanked for their participation. Each session took approximately an hour.

### Content coding

Based on the video, a detailed coding scheme was created. Throughout the coding process, the scheme was expanded with new details mentioned by participants that were not in the original coding scheme. The final coding scheme contained 312 details. Each detail could be coded as correct (e.g., “his hair was blonde”), incorrect (e.g., “his hair was black”) or subjective (e.g., “his hair was beautiful”). To determine the accuracy of measurable descriptive details about the man and the woman in the video (e.g., age, height, weight), we contacted the actors in the video, who kindly provided us with the relevant information. Answers were counted as correct if they were in the range of five years younger or older, 5 cm shorter or taller and 5 k lighter or heavier than the details provided by the actors. To determine the accuracy of non-measurable descriptive details (e.g., hair color, hair style, facial hair, clothing), we conducted pilot testing. We showed ten pilot participants the video and asked them to describe the appearance and clothing of the man and the woman. Based on their descriptions, we determined which answers should be counted as correct. For example, the man's hair was described as “blonde” or “light” by all ten pilot participants, so only “blonde” or “light” were counted as correct answers. The woman's hair, on the other hand, was alternately described as brown (2 participants), red (2 participants), brown/red, reddish, red/orange, orange, dark, and brown/dark. In the main study, we counted all of those answers as correct. The pilot participants did not participate in the main study.

Two independent coders blind to the study's hypotheses each coded half of the interviews based on the audio-recordings. For each of the 312 details, the coder scored whether it had been mentioned correctly, incorrectly, both correctly and incorrectly (at different points in the interview), subjectively, or not at all. In addition, both coders double-coded 12% of the data (i.e., all interviews of 18 randomly selected pairs, 33,696 data points). Interrater reliability was high (percentage agreement = 97%; κ = 0.88, *p* < 0.001; κ maximum = 0.98). The scores of the original coder were retained for the main analysis.

### Retrieval strategy coding

To analyse the collaborative retrieval strategies used by witness pairs, all collaborative interviews were transcribed verbatim. Transcripts were coded based on the retrieval strategy coding scheme used by Vredeveldt et al. (2016a), with the addition of one category: checking accuracy (see also Vredeveldt et al., 2016b). Table 1 displays definitions, examples and observed frequencies for each retrieval strategy in the coding scheme. Each statement in the transcript could be coded as one of the 13 retrieval strategies listed in Table 1 or as “no strategy.” Because coding retrieval strategies is not as straightforward as content coding, all transcripts were coded by two independent coders, blind to the study's hypotheses. Interrater reliability was acceptable (percentage agreement = 83%; κ = 0.69, *p* < 0.001; κ maximum = 0.94). Disagreements were resolved by discussion and the agreed-upon codes were retained for the analysis.

**Table 1:** Retrieval strategy coding categories with descriptions and examples.

**Strategy**	**Description and examples**	**No instruction**		**Instruction**	
		***M***	***SD***	***M***	***SD***
Successful cue^b^	Cuing attempt (e.g., “What was he wearing?”) that is followed by retrieval of information by the partner (e.g., “Jeans”).	0.72	0.61	1.64	1.75
Failed cue	Cuing attempt (e.g., “What was he wearing?”) that is not followed by retrieval of information by the partner (e.g., “I don't remember”).	1.32	1.31	1.84	1.65
Acknowledgment	Indicating support for a partner's statement, such as “Yes”, “Yeah”, “Hm hm”, or “That's right”.	32.92	20.94	36.40	16.56
Correction	Correcting a partner's statement (e.g., “No, it was shorts”), or questioning its accuracy (e.g., “I remember it differently”).	10.84	2.59	1.72	1.40
Elaboration	Building on a partner's statement by providing additional information, either countable (i.e., a new detail as classified in the content coding scheme) or non-countable (e.g., “she looked creepy”).	3.76	3.50	3.44	30.14
Explanation^b^	Explaining one's own statement to the partner (e.g., “I remember thinking it looked too cold for shorts.”).	1.84	1.82	1.32	1.18
Repetition	Repeating a partner's statement verbatim.	4.08	2.81	4.96	3.95
Restatement	Reformulating a partner's statement without changing the content (e.g., rephrasing “jeans” to “denim trousers”).	3.00	2.35	2.28	1.54
Renewed remembering	Indicating that a partner's statement triggers a memory (e.g., “Now I remember it again” or “I had forgotten about that!”).	1.36	1.55	1.92	1.61
Positive references to relationship^a^	Positive statement about the partner's or the pair's ability (e.g., “I am impressed that you remember that” or “We remember this quite well”).	0.08	0.28	0.24	0.44
Negative references to relationship^a^	Negative statement about the partner's or the pair's ability (e.g., “You have such bad memory” or “We are probably wrong about this”).	0.40	0.91	0.28	0.54
Role division^a^	Dividing or organizing the retrieval task (e.g., “Do you want to start?” or “You describe him, and I'll add to your description”).	0.92	1.12	0.4	0.76
Checking accuracy	Checking with the partner whether particular details are correct (e.g., “He was wearing jeans, right?”).	1.44	1.42	1.96	2.15
Total number of strategies		53.68	30.78	58.4	20.18

*Means (M) and standard deviations (SD) for the frequency of occurrence per collaborative interview in the collaborative-none and the collaborative-instruction conditions (adapted from Vredeveldt et al., 2016a)*.

a *Not included in parametric analyses and principal component analysis because it occurred less than once per interview on average*.

b *Not included in principal component analysis because eight out of nine correlations with other strategies were below 0.3*.

## Results

In dyadic data analysis, if there is any indication of non-independence, then pair performance should be used as unit of analysis rather than individual performance (Kenny et al., 2006). Therefore, we first computed partial intraclass correlations for the pair members' correct and incorrect recall scores in each interview, taking into account variation as a result of experimental condition (i.e., a hierarchically nested design). The partial intraclass correlation for incorrect recall at Interview 3 (*r* = 0.21, *p* = 0.065) was significant at the liberal α-level of 0.20 recommended for tests of non-independence (Myers, 1979; Kenny et al., 2006). In other words, there was an indication of non-independence. We therefore entered pair performance as the dependent variable in all analyses reported in the manuscript. This analytical approach is also the most relevant from a practical perspective, given that we are interested in the quantity and quality of the total amount of information that can be obtained from a pair of witnesses.

Specifically, the analyses for all recall measures reflect the number of non-redundant details obtained per witness pair (i.e., the same detail mentioned by both pair members is counted only once). Items described both correctly and incorrectly in the same interview (e.g., the man's hair is first described as black but later as blonde) counted toward the number of correct details as well as the number of incorrect details. Subjective details did not count toward correct or incorrect details. Prior to analysis, all relevant assumptions were checked and transformations were applied where necessary (as explained in more detail in the relevant sections below). All reported *p*-values are two-tailed. In line with the Open Science Framework guidelines, we report all analyses that were preregistered and identify any analyses that were not preregistered.

### Manipulation check

Table 1 shows how frequently each retrieval strategy occurred during the collaborative interviews for pairs who had been instructed on how to collaborate effectively (collaborative-instruction) and for pairs who had not been instructed (collaborative-none). The prerequisite assumption for our prediction that collaborative-instruction pairs would report more information than collaborative-none pairs, was that they would follow the instructions. The first step was therefore to check whether pairs in the collaborative-instruction condition used more or different retrieval strategies than pairs in the collaborative-none condition (i.e., a manipulation check, which was not preregistered). In terms of the total number of strategies, there was no significant difference between conditions (see Table 1), *F*_(1, 48)_ = 1.10, *p* = 0.300, η^2^ = 0.02.

In addition to the total number of retrieval strategies used, we also assessed what type of strategies pair members used during the discussion. Before entering each type of strategy in a multivariate analysis of variance (MANOVA), we removed positive and negative references to the relationship and role division, because they occurred less than once per interview on average (see Table 1). The distributions for the remaining retrieval strategies were all significantly positively skewed and some were leptokurtic. Square-root transformation of the raw frequencies solved all problems with non-normality. A MANOVA with the square-root transformed frequencies of successful cues, failed cues, acknowledgments, corrections, elaborations, explanations, repetitions, restatements, remembers again and checking accuracy as dependent variables revealed no significant multivariate effect, *F*_(10, 39)_ = 1.65, *p* = 0.129, η^2^ = 0.30. None of the simple effects were significant (all *p*s > 0.088, all η^2^s < 0.06).

Finally, there was no significant difference in interview duration between collaborative pairs who received instructions (*M* = 10.74 min, *SD* = 2.50) and collaborative pairs who did not receive instructions (*M* = 9.89 min, *SD* = 2.97), *t*_(48)_ = 1.09, *p* = 0.281, *d* = 0.31.^1^ In sum, the instruction to use specific collaborative strategies had no significant effect on the number or type of strategies that pairs used during the collaborative interview, nor on how long the interview took to complete. The strategy instruction was not successful at encouraging a more content-focused interaction style.

### Correct recall

#### Information per interview

**Figure 1:**
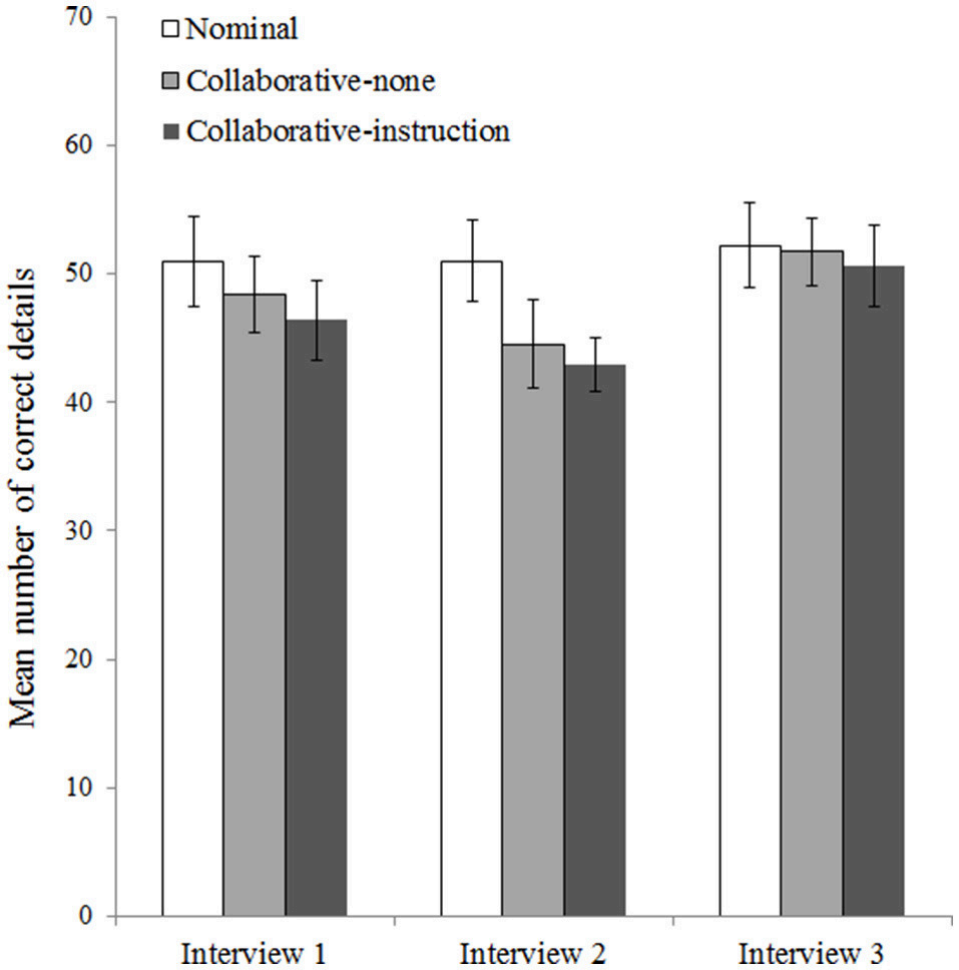
Mean number of correct details mentioned by pairs in the nominal (*n* = 25), collaborative-none (*n* = 25), and collaborative-instruction (*n* = 25) conditions during the first, second, and third interview. Error bars represent 95% confidence intervals.

To analyse the number of correct details each pair reported per interview, we conducted a 3 (Condition: nominal, collaborative-none, collaborative-instruction) × 3 (Interview: 1, 2, 3) mixed ANOVA. We found significant main effects of condition, *F*_(2, 72)_ = 3.22, *p* = 0.046, η^2^ = 0.08, and interview, *F*_(2, 144)_ = 36.91, *p* < 0.001, η^2^ = 0.31, and a significant interaction between condition and interview, *F*_(4, 144)_ = 5.69, *p* < 0.001, η^2^ = 0.09. Figure 1 shows the interaction pattern.

The effect of interview was not significant for the nominal condition, *F*_(2, 71)_ = 0.98, *p* = 0.380, η^2^ = 0.03, but it was significant for the collaborative-none condition, *F*_(2, 71)_ = 20.10, *p* < 0.001, η^2^ = 0.36, and the collaborative-instruction condition, *F*_(2, 71)_ = 23.42, *p* < 0.001, η^2^ = 0.40. The effect of condition was not significant for the first interview, *F*_(2, 72)_ = 2.19, *p* = 0.119, η^2^ = 0.06, or the third interview, *F*_(2, 72)_ = 0.32, *p* = 0.725, η^2^ = 0.01, but it was significant for the second interview, *F*_(2, 72)_ = 8.90, *p* < 0.001, η^2^ = 0.20. We examined the significant effect of condition during Interview 2 (see Figure 1) with three simple ANOVAs (Bonferroni-corrected α = 0.017). Pairs in the nominal condition reported significantly more correct details in Interview 2 than pairs in the collaborative-none condition, *F*_(1, 48)_ = 8.15, *p* = 0.006, η^2^ = 0.15, and pairs in the collaborative-instruction condition, *F*_(1, 48)_ = 19.07, *p* < 0.001, η^2^ = 0.28. The two collaborative conditions did not differ significantly, *F*_(1, 48)_ = 0.68, *p* = 0.414, η^2^ = 0.01.

#### New information

When repeated interviews are conducted with witnesses, the most relevant question from a practical perspective is how much *new* information can be obtained in later interviews. We first examined what percentage of correct information reported during Interview 2 was new (i.e., not reported by either of the pair members in Interview 1). There was a significant effect of condition, *F*_(2, 72)_ = 3.64, *p* = 0.031, η^2^ = 0.09, which was examined further with three simple ANOVAs (Bonferroni-corrected α = 0.017). Of the correct information reported in the second interview, 16% was new (*SD* = 7%) for pairs in the collaborative-instruction condition, 13% (*SD* = 6%) in the collaborative-none condition and 12% in the nominal condition (*SD* = 6%). Collaborative-instruction pairs reported a significantly higher percentage of new information than nominal pairs, *F*_(1, 48)_ = 6.76, *p* = 0.012, η^2^ = 0.12. Non-significant differences were observed between the nominal and collaborative-none conditions, *F*_(1, 48)_ = 0.67, *p* = 0.418, η^2^ = 0.01, and between the two collaborative conditions, *F*_(1, 48)_ = 3.20, *p* = 0.080, η^2^ = 0.06.

We also examined what percentage of correct information reported during Interview 3 was new (i.e., not reported by either of the pair members in Interview 1 or 2). The effect of condition was not significant, *F*_(2, 72)_ = 2.94, *p* = 0.059, η^2^ = 0.08, but the observed data pattern was in the same direction as for Interview 2: pairs in the collaborative-instruction reported 10% new correct information on average (*SD* = 5%), pairs in the collaborative-none condition reported 9% new correct information (*SD* = 6%) and pairs in the nominal condition reported 7% new correct information (*SD* = 4%).

#### Omitted information

Next, we assessed what percentage of correct information reported in earlier interviews was not mentioned again in later interviews. For the percentage of omitted information in Interview 2, there was a large and significant effect of condition, *F*_(2, 72)_ = 15.91, *p* < 0.001, η^2^ = 0.31. Pairs in the nominal condition omitted only 11% of previously reported correct information (*SD* = 5%), whereas pairs in both collaborative conditions omitted 21% (*SD* = 8%). Three simple ANOVAs (Bonferroni-corrected α = 0.017) revealed that the nominal condition differed significantly from the collaborative-none condition, *F*_(1, 48)_ = 25.24, *p* < 0.001, η^2^ = 0.34, and from the collaborative-instruction condition, *F*_(1, 48)_ = 27.84, *p* < 0.001, η^2^ = 0.37, but there was no difference between the two collaborative conditions, *F*_(1, 48)_ = 0.07, *p* = 0.797, η^2^ = 0.00.

For the percentage of omitted correct information in Interview 3, there was no significant difference between conditions, *F*_(2, 72)_ = 0.31, *p* = 0.736, η^2^ = 0.01.

#### Total number of correct details

The analyses reported above show that pairs in both collaborative conditions suffered from collaborative inhibition during Interview 2. In a police interview setting, however, it is more relevant to know whether collaboration reduces the total amount of correct information obtained from a pair of witnesses across all interviews. That is, the reduced amount of information reported by collaborative pairs in Interview 2 may solely be due to the omission of information that was reported in one of the other interviews and may thus not affect the overall amount of information obtained from a witness pair. We therefore examined the total amount of non-redundant correct information reported across interviews (i.e., the same detail mentioned in multiple interviews is counted only once).

There was no significant effect of condition on the number of non-redundant correct details reported across all interviews, *F*_(2, 72)_ = 0.51, *p* = 0.601, η^2^ = 0.01. Nominal pairs provided 62.04 correct details overall (*SD* = 9.25), collaborative-none pairs provided 60.36 details (*SD* = 7.63) and collaborative-instruction pairs provided 59.76 details (*SD* = 7.79). Thus, the individual interviews before and after the collaborative interview compensated for the inhibitory effects associated with collaborative recall.

To examine whether conducting just one individual interview, prior to collaboration, would be sufficient to compensate for collaborative inhibition during Interview 2, we conducted an exploratory analysis of the total number of non-redundant correct details reported by pairs in the first two interviews (i.e., ignoring Interview 3). Again, we found no significant effect of condition, *F*_(2, 72)_ = 1.43, *p* = 0.246, η^2^ = 0.04. Nominal pairs provided 57.64 correct details in the first two interviews (*SD* = 9.04), collaborative-none pairs 55.04 (*SD* = 7.83) and collaborative-instruction pairs 53.92 (*SD* = 6.92). Thus, the inhibitory effects of collaboration on the amount of information reported can be overcome by interviewing witnesses individually before they collaborate. Two witnesses who are interviewed individually and then collaboratively (with or without instructions) provide just as much correct information overall as two witnesses who are interviewed individually twice.

### Incorrect recall

Prior to the analysis of incorrect details, square-root transformations were applied to the raw frequencies, which alleviated problems with positive skew and leptokurtosis.

#### Errors per interview

**Figure 2:**
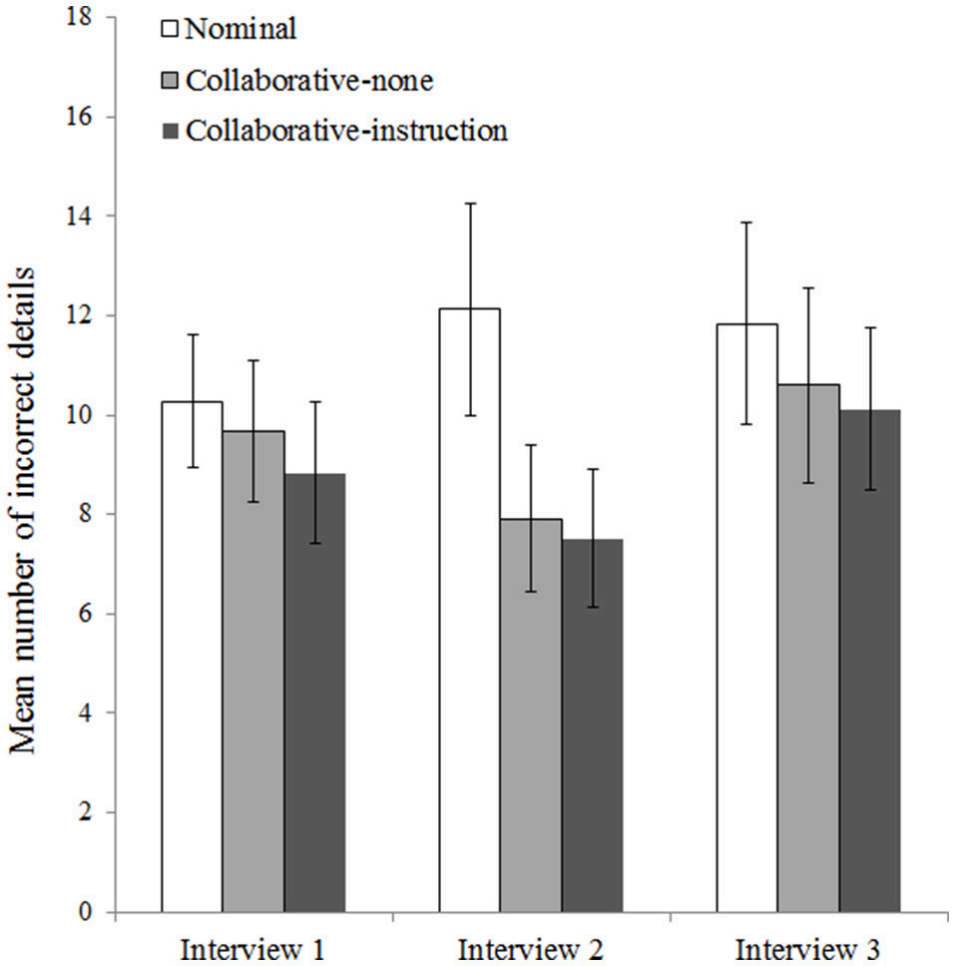
Mean number of incorrect details mentioned by pairs in the nominal (*n* = 25), collaborative-none (*n* = 25), and collaborative-instruction (*n* = 25) conditions during the first, second, and third interview. Error bars represent 95% confidence intervals.

To analyse the number of incorrect details reported per interview, we conducted a 3 (Condition: nominal, collaborative-none, collaborative-instruction) × 3 (Interview: 1, 2, 3) mixed ANOVA on the square-root transformed number of errors. We found a significant effect of condition, *F*_(2, 72)_ = 3.54, *p* = 0.034, η^2^ = 0.09, a significant effect of interview, *F*_(2, 144)_ = 13.08, *p* < 0.001, η^2^ = 0.13, and a significant interaction between condition and interview, *F*_(4, 144)_ = 6.20, *p* < 0.001, η^2^ = 0.13. Figure 2 shows the interaction pattern.

The effect of interview was not significant for the nominal condition, *F*_(2, 71)_ = 2.93, *p* = 0.060, η^2^ = 0.08, but it was significant for the collaborative-none condition, *F*_(2, 71)_ = 12.17, *p* < 0.001, η^2^ = 0.26, and the collaborative-instruction condition, *F*_(2, 71)_ = 14.07, *p* < 0.001, η^2^ = 0.28. The effect of condition was not significant for the first interview, *F*_(2, 72)_ = 1.27, *p* = 0.286, η^2^ = 0.03, or the third interview, *F*_(2, 72)_ = 0.92, *p* = 0.405, η^2^ = 0.02, but it was significant for the second interview, *F*_(2, 72)_ = 9.13, *p* < 0.001, η^2^ = 0.20. Three simple ANOVAs (Bonferroni-corrected α = 0.017) to break down the significant effect at Interview 2 revealed that pairs in the nominal condition made significantly more errors during the second interview than pairs in the collaborative-none condition, *F*_(1, 48)_ = 11.66, *p* = 0.001, η^2^ = 0.20, and pairs in the collaborative-instruction condition, *F*_(1, 48)_ = 14.67, *p* < 0.001, η^2^ = 0.23 (see Figure 2). The two collaborative conditions did not differ significantly, *F*_(1, 48)_ = 0.14, *p* = 0.713, η^2^ = 0.00.

#### New errors

We examined whether the conditions differed in what percentage of the reported incorrect information was new. For Interview 2, there was no significant difference between conditions, *F*_(2, 72)_ = 0.15, *p* = 0.863, η^2^ = 0.00. Of the errors made in the second interview, 21% (*SD* = 18%) was new for nominal pairs, compared to 21% (*SD* = 18%) for collaborative-none pairs and 24% (*SD* = 20%) for collaborative-instruction pairs.

We also examined what percentage of errors in Interview 3 was new. Again, condition had no significant effect, *F*_(2, 72)_ = 2.27, *p* = 0.110, η^2^ = 0.06. Of the errors made in the third interview, 11% (*SD* = 10%) was new in the nominal condition, 16% (*SD* = 12%) in the collaborative-none condition and 17% (*SD* = 12%) in the collaborative-instruction condition.

#### Omitted errors

Next, we assessed what percentage of errors made in earlier interviews was omitted in later interviews. For Interview 2, we found a significant effect of condition, *F*_(2, 72)_ = 3.59, *p* = 0.032, η^2^ = 0.09. Pairs in the collaborative-none condition purged the highest percentage of previously made errors from their testimony in the second interview (*M* = 30%; *SD* = 19%), followed by pairs in the collaborative-instruction condition (*M* = 26%; *SD* = 16%). The lowest percentage of omitted errors in Interview 2 was observed for pairs in the nominal condition (*M* = 18%; *SD* = 13%). Three simple ANOVAs (Bonferroni-corrected α = 0.017) showed that only the difference between the collaborative-none condition and the nominal condition was significant, *F*_(1, 48)_ = 7.02, *p* = 0.011, η^2^ = 0.13. Pairs in the collaborative-instruction condition did not differ significantly from the nominal condition, *F*_(1, 48)_ = 3.47, *p* = 0.069, η^2^ = 0.07, or the collaborative-none condition, *F*_(1, 48)_ = 0.77, *p* = 0.385, η^2^ = 0.02.

We found no significant effect of condition on the percentage of omitted errors in Interview 3, *F*_(2, 72)_ = 0.91, *p* = 0.406, η^2^ = 0.02.

#### Total number of errors

Collaborative pairs (regardless of instruction) made significantly fewer errors during Interview 2 than nominal pairs, but from a practical perspective, we also want to know whether experimental condition affected the total number of non-redundant errors that pairs reported across interviews (i.e., the same error mentioned in multiple interviews is counted only once). Across all three interviews, nominal pairs reported 16.40 non-redundant errors (*SD* = 5.63), collaborative-none pairs 14.88 (*SD* = 5.73) and collaborative-instruction pairs 14.40 (*SD* = 5.13). The difference between conditions was not significant, *F*_(2, 72)_ = 0.95, *p* = 0.390, η^2^ = 0.03.

We also conducted an exploratory analysis of the total number of non-redundant errors reported in the first two interviews only. For this measure, there was a significant effect of condition, *F*_(2, 72)_ = 3.43, *p* = 0.038, η^2^ = 0.09. Nominal pairs made 14.68 errors (*SD* = 5.22) in the first two interviews, collaborative-none pairs 12.04 (*SD* = 4.25) and collaborative-instruction pairs 11.44 (*SD* = 4.32). Thus, the testimony provided by collaborative pairs in the first two interviews contained fewer errors than the testimony provided by nominal pairs.

We followed up the significant effect of condition on the number of non-redundant errors reported in the first two interviews with three simple ANOVAs (Bonferroni-corrected α = 0.017). The difference between the nominal condition and the collaborative-instruction condition just failed to reach significance at the Bonferroni-corrected level, *F*_(1, 48)_ = 5.91, *p* = 0.019, η^2^ = 0.11. The collaborative-none condition did not differ significantly from either the nominal condition, *F*_(1, 48)_ = 3.89, *p* = 0.054, η^2^ = 0.07, or the collaborative-instruction condition, *F*_(1, 48)_ = 0.28 *p* = 0.598, η^2^ = 0.01.

### Retrieval strategies

Previous studies have found that the retrieval strategies listed in Table 1 can be categorized into two distinct interaction styles: content-focused interaction and process-focused interaction. The first style is characterized by a focus on the content of the to-be-remembered information and encompasses the strategies to acknowledge, repeat, rephrase and elaborate upon each other's contributions. This interaction style has been found to positively predict the amount of information reported by pairs of witnesses. The second interaction style is somewhat more diverse, but focuses primarily on the process of remembering together. It encompasses the strategies of explanations, corrections, role division, successful and failed cuing attempts, positive and negative references to the relationship and expressions of renewed remembering. To find out how the new strategy we added to the coding scheme, checking accuracy, would fit into the existing framework, we conducted a principal components analysis on the retrieval strategies used by pairs in both collaborative conditions (*N* = 50 pairs).

Prior to the principal components analysis, we removed successful cues and explanations, because they did not correlate with the other strategies; eight out of nine correlations were below 0.3 (see Field, 2013). A principal components analysis with direct oblimin rotation on the square-root transformed frequencies of retrieval strategies revealed two components with an Eigenvalue greater than 1, which together explained 58.1% of the variance. Table 2 shows the loadings of each strategy onto the components. The first component (α = 0.72) was identical to the content-focused interaction component identified by Vredeveldt et al. (2016a). The second component (α = 0.69) was closely related to Vredeveldt et al.'s process-focused interaction component. The newly added strategy, checking accuracy, fit within the latter component.

**Table 2:** Oblimin-rotated pattern matrix from the principal components analysis showing content-focused interaction (α = 0.72) and process-focused interaction (α = 0.69).

	**Component**	
**Variable**	**Content-focused**	**Process-focused**
Acknowledgment	0.799	
Restatement	0.781	
Repetition	0.669	
Elaboration	0.621	
Checking accuracy		0.856
Remembers again		0.628
Correction		0.626
Failed cue		0.568

*Variables are sorted according to the size of their contribution. Loadings below 0.3 are not depicted*.

We conducted a linear regression analysis to investigate whether the two types of interaction styles were associated with the amount of information reported during collaborative interviews (i.e., total number of correct and incorrect details). The model with both interaction component scores as predictors explained a significant proportion of the variance in the amount of information reported per pair during the collaborative interview, *R*^2^ = 0.14, *F*_(2, 47)_ = 3.78, *p* = 0.030. The content-focused interaction component significantly predicted the amount reported, β = 0.29, *t*_(49)_ = 2.05, *p* = 0.046, whereas the process-focused interaction component did not, β = 0.17, *t*_(49)_ = 1.18, *p* = 0.246.

We also assessed whether the interaction styles predicted the accuracy of information reported during collaborative interviews (i.e., the number of correct details divided by the total number of correct and incorrect details). The model with both interaction components did not explain a significant proportion of the variance in accuracy, *R*^2^ = 0.04, *F*_(2, 47)_ = 1.08, *p* = 0.349.

Thus, we found support for our prediction that pairs who acknowledge, repeat, rephrase, and elaborate upon each other's contributions would report more information during the collaborative interview. A qualitative example of that content-focused interaction style is provided below.

A: I heard daunting music in the background
*B: Oh yes, me too. Nice chase music*.
A: I also remember the sound of the car
*B: Yes, definitely the car. You could hear the engine revving the whole time*.
*A: Yes*.
*B: And I don't remember them talking much, maybe just a little like “go here, go there” or something but I'm not 100% sure*.
*A: Yeah I think that's right*.

In this example, witness A comments on the background music (“daunting music”) and witness B acknowledges and rephrases her statement (“chase music”). Witness A then mentions another sound she heard (“the car”), which witness B acknowledges, repeats and elaborates upon (“engine revving”). Witness A acknowledges that elaboration. Witness B then discusses what was said in the video, which is acknowledged again by Witness A. This example illustrates how witnesses can feed off of each other's statements during a collaborative interview and remember more together, provided that they communicate effectively (for more examples, see Vredeveldt et al., 2016a, 2017).

## Discussion

In the present study, we examined how collaboration with or without strategy instructions affected the amount of correct and incorrect information reported by pairs of witnesses. Our strategy instruction did not have a significant effect on the number or type of retrieval strategies used during the collaborative interview, and did not affect memory performance. We found that collaborative pairs reported less correct information than nominal pairs during the second interview (i.e., collaborative inhibition), but that was solely due to the omission of information that had already been reported in the first interview. Moreover, collaborative pairs reported significantly fewer errors than nominal pairs (i.e., error pruning), not only during the collaborative interview itself but also across the first two interviews. Finally, we replicated previous findings that pairs who acknowledged, repeated, rephrased and elaborated upon each other's contributions remembered more together.

The main goal of the present study was to explore whether instructions on how to collaborate can enhance the memory performance of pairs of witnesses. Our strategy instructions, however, had no effect on the number or type of collaborative strategies used by witness pairs. It therefore came as no surprise that the instructions did not affect memory performance either. But why did our strategy instructions not affect the retrieval strategies used during the collaborative interview? One potential explanation could be that participants who do not listen carefully to their partner's contributions during the discussion (i.e., do not adopt a content-focused interaction style), also do not listen carefully to the interviewer's instructions. In that case, instructing those participants on how to collaborate is unlikely to have an effect. Conversely, participants who do listen carefully to the interviewer's instructions, are probably already inclined to listen carefully to the partner's contributions as well. In other words, the type of participant who complies with the instructions may be more likely to use content-focused strategies anyway, without prompting by the interviewer.

Future studies should explore whether the observed null effects of strategy instruction were due to the nature of the instruction used in the present study, or due to the fact that effective collaboration strategies in witness interviews simply cannot be taught (i.e., it is possible that the content-focused interaction style is only successful if witnesses adopt it spontaneously). Future researchers may want to implement strategy instructions more forcefully, for example by asking participants to repeat the instructions back to them before starting the collaborative interview or by introducing a practice round with feedback from the interviewer, who could intervene when one participant does not repeat and elaborate upon the partner's contributions. Another method worth exploring would be to provide witnesses with a model statement or model video of a successful collaborative interaction (cf. Leal et al., 2015; Brackmann et al., 2017; Vrij et al., 2017).

Compared to previous studies (Vredeveldt et al., 2016a,b, 2017), pairs in this study used relatively few retrieval strategies during the collaborative interview. That was probably due to the fact that they were interviewed about a short and simple event (a 70-s video clip of a car chase), whereas pairs in previous studies were interviewed about more complicated and longer events (an 8-min video clip featuring various conversations, shoot-outs and physical fights; Vredeveldt et al., 2017; a 5-min scene in a play in which a man gets murdered and a woman gets raped, Vredeveldt et al., 2016a; and an elaborate live police training exercise involving the arrest of a suspicious man in a car, Vredeveldt et al., 2016b). Nevertheless, just like in previous studies, content-focused strategies in the present study were significantly positively associated with the amount of information reported by pairs of witnesses. This is a remarkably consistent finding across studies, particularly in light of the fact that witnesses in the present study were complete strangers to each other before the experiment. Apparently, the combination of acknowledge-repeat-rephrase-elaborate is an effective communication style for long-time married couples, university friends, police partners, and strangers alike. A limitation of the sample in the present study was the significant age difference between the collaborative-none condition and the other two conditions. We do not believe this age difference would have affected the results, partly because the difference was due to a relatively low number of older participants in the other two conditions that resulted in an extremely skewed distribution, and partly because previous studies have reported the same pattern of collaborative effects with samples that varied widely in age. Nonetheless, to exclude possible influences of age, future studies should ensure that participant samples in all conditions are equivalent in terms of age and other demographic variables.

During the second interview, we found evidence for both collaborative inhibition and error pruning. The total amount of correct and incorrect information obtained across all three interviews, however, did not differ between conditions. Thus, conducting an individual interview before and after the collaborative interview compensates for the inhibitory effects of collaboration. From a practical perspective, conducting an individual interview prior to collaboration makes sense because it allows the police to identify the original source of reported information. The practical value of an individual interview *after* collaboration, however, is not immediately apparent. We therefore explored what happens if we ignore the information obtained in the third interview. We found no differences between conditions in the amount of correct information gathered across the first two interviews. Thus, a single individual interview prior to collaboration was sufficient to protect against collaborative inhibition. Moreover, there was a significant effect of condition on the number of errors reported across the first two interviews, with collaborative pairs making fewer errors than nominal pairs. In sum, our findings show that the procedure of conducting an individual interview prior to a collaborative interview eliminates collaborative inhibition while maintaining error pruning benefits.

In conclusion, based on our findings in combination with those of previous studies, we can provide some tentative recommendations to police interviewers. First, our exploratory analysis of information collected across the first two interviews showed that the combination of an individual and a collaborative interview, as compared to two individual interviews, resulted in the elicitation of just as much correct information but fewer errors. This replicates previous findings (Vredeveldt et al., 2016a, 2017). Thus, when police interviewers have access to a pair of witnesses, it may be best to first conduct an individual interview with each witness and then interview them together. An additional benefit of that procedure, crucial in legal settings, is that it allows police interviewers to obtain an independent account from each witness before they can influence each other. Second, although our findings replicate Vredeveldt and colleagues' findings that the use of content-focused retrieval strategies during the collaborative interview is associated with a greater amount of information reported, it seems that instructing witnesses to use those strategies may not improve recall output. Our instructions on how to collaborate effectively did not affect the witnesses' use of retrieval strategies, nor how much or how accurately they remembered. This points to the possibility that successful collaborative strategies cannot be taught. Perhaps, when it comes to effective collaboration, you either have it or you don't.

## Author contributions

AV: designed the study, organized the data collection, performed the data analysis, and drafted the manuscript; PvK: contributed to the study design and advised during all stages of the project; Both authors approved the final version of the manuscript for submission.

### Conflict of interest statement

The authors declare that the research was conducted in the absence of any commercial or financial relationships that could be construed as a potential conflict of interest.

## Footnotes

^1^Individual interviews at Time 2 took less time on average (*M* = 6.88 minutes, *SD* = 1.95) than collaborative interviews, but conducting two individual interviews for pairs in the nominal condition took significantly longer (*M* = 13.75 minutes, *SD* = 2.84) than conducting a single collaborative interview for pairs in the collaborative-none condition, *t* (48) = 4.70, *p* < 0.001, *d* = 1.33, and the collaborative-instruction condition, *t* (48) = 3.98, *p* < 0.001, *d* = 1.13.

## References

[B1] Barber S. J. Harris C. B. Rajaram S. (2015). Why two heads apart are better than two heads together: multiple mechanisms underlie the collaborative inhibition effect in memory. J. Exp. Psychol. 41, 559–566. 10.1037/xlm0000037 25068855 PMC4309738

[B2] Bärthel G. A. Wessel I. Huntjens R. J. Verwoerd J. (2017). Collaboration enhances later individual memory for emotional material. Memory 25, 636–646. 10.1080/09658211.2016.1208248 27403926

[B3] Basden B. H. Basden D. R. Bryner S. Thomas R. L. III. (1997). A comparison of group and individual remembering: does collaboration disrupt retrieval strategies? J. Exp. Psychol. 23, 1176–1189. 10.1037/0278-7393.23.5.1176 9293628

[B4] Brackmann N. Otgaar H. Roos af Hjelmsäter E. Sauerland M. (2017). Testing a new approach to improve recall in different ages: providing witnesses with a model statement. Transl. Issues Psychol. Sci. 3, 131–142. 10.1037/tps0000116

[B5] Field A. P. (2013). Discovering Statistics using SPSS, 4th Edn. London: Sage.

[B6] Gabbert F. Memon A. Allan K. (2003). Memory conformity: can eyewitnesses influence each other's memories for an event? Appl. Cogn. Psychol. 17, 533–543. 10.1002/acp.885

[B7] Harris C. B. Barnier A. J. Sutton J. (2012). Consensus collaboration enhances group and individual recall accuracy. Q. J. Exp. Psychol. 65, 179–194. 10.1080/17470218.2011.608590 21939368

[B8] Harris C. B. Barnier A. J. Sutton J. Keil P. G. (2014). Couples as socially distributed cognitive systems: remembering in everyday social and material contexts. Mem. Stud. 7, 285–297. 10.1177/1750698014530619

[B9] Harris C. B. Keil P. G. Sutton J. Barnier A. J. McIlwain D. J. F. (2011). We remember, we forget: collaborative remembering in older couples. Discourse Process. 48, 267–303. 10.1080/0163853X.2010.541854

[B10] Hyman I. E. Jr. Cardwell B. A. Roy R. A. (2013). Multiple causes of collaborative inhibition in memory for categorised word lists. Memory 21, 875–890. 10.1080/09658211.2013.769058 23439153

[B11] Kenny D. A. Kashy D. A. Cook W. L. (2006). Dyadic Data Analysis. New York, NY: Guilford Press.

[B12] Leal S. Vrij A. Warmelink L. Vernham Z. Fisher R. P. (2015). You cannot hide your telephone lies: providing a model statement as an aid to detect deception in insurance telephone calls. Legal Criminol. Psychol. 20, 129–146. 10.1111/lcrp.12017

[B13] Marion S. B. Thorley C. (2016). A meta-analytic review of collaborative inhibition and post-collaborative memory: a test of the retrieval disruption hypothesis. Psychol. Bull. 142, 1141–1164. 10.1037/bul0000071 27618544

[B14] Meade M. L. Nokes T. J. Morrow D. G. (2009). Expertise promotes facilitation on a collaborative memory task. Memory 17, 39–48. 10.1080/09658210802524240 19105086

[B15] Meade M. L. Roediger H. L. III. (2002). Explorations in the social contagion of memory. Mem. Cognit. 30, 995–1009. 10.3758/BF03194318 12507365

[B16] Myers J. L. (1979). Fundamentals of Experimental Design, 3rd Edn. Boston, MA: Allyn & Bacon.

[B17] Roediger H. L. III. Meade M. L. Bergman E. (2001). Social contagion of memory. Psychon. Bull. Rev. 8, 365–371. 10.3758/BF03196174 11495127

[B18] Ross M. Spencer S. J. Linardatos L. Lam K. C. H. Perunovic M. (2004). Going shopping and identifying landmarks: does collaboration improve older people's memory? Appl. Cogn. Psychol. 18, 683–696. 10.1002/acp.1023

[B19] Shaw D. J. Vrij A. Leal S. Mann S. A. Hillman J. Granhag P. A. (2014). ‘We'll take it from here’: the effect of changing interviewers in information gathering interviews. Appl. Cogn. Psychol. 28, 908–916. 10.1002/acp.3072

[B20] Van Amelsvoort A. Rispens I. Grolman H. (2015). Handleiding Verhoor, 6th Edn. Den Haag: Elsevier Overheid.

[B21] Vredeveldt A. Groen R. N. Ampt J. E. van Koppen P. J. (2017). When discussion between eyewitnesses helps memory. Legal Criminol. Psychol. 22, 242–259. 10.1111/lcrp.12097

[B22] Vredeveldt A. Hildebrandt A. Van Koppen P. J. (2016a). Acknowledge, repeat, rephrase, elaborate: witnesses can help each other remember more. Memory 24, 669–682. 10.1080/09658211.2015.1042884 26299652

[B23] Vredeveldt A. Kesteloo L. Van Koppen P. J. (2016b). Samen of apart: de invloed van overleg tussen agenten tijdens het opstellen van het proces-verbaal. [together or apart: the influence of discussion between police officers while writing incident reports]. Apeldoorn: Programma Politie & Wetenschap.

[B24] Vrij A. Leal S. Mann S. Dalton G. Jo E. Shaboltas A.. (2017). Using the model statement to elicit information and cues to deceit in interpreter-based interviews. Acta Psychol. 177, 44–53. 10.1016/j.actpsy.2017.04.011 28477454

[B25] Weldon M. S. Bellinger K. D. (1997). Collective memory: collaborative and individual processes in remembering. J. Exp. Psychol. 23, 1160–1175. 10.1037/0278-7393.23.5.1160 9293627

[B26] Wessel I. Zandstra A. R. Hengeveld H. M. Moulds M. L. (2015). Collaborative recall of details of an emotional film. Memory 23, 437–444. 10.1080/09658211.2014.895384 24628679

[B27] Wright D. B. Self G. Justice C. (2000). Memory conformity: exploring misinformation effects when presented by another person. Br. J. Psychol. 91, 189–202. 10.1348/000712600161781 10832514

[B28] Yaron-Antar A. Nachson I. (2006). Collaborative remembering of emotional events: the case of Rabin's assassination. Memory 14, 46–56. 10.1080/09658210444000502 16423741

